# Wireless Measuring System for Monitoring the Condition of Devices Designed to Protect Line Structures †

**DOI:** 10.3390/s20092512

**Published:** 2020-04-29

**Authors:** Martin Pieš, Radovan Hájovský, Jan Velička

**Affiliations:** Department of Cybernetics and Biomedical Engineering, VSB-Technical University of Ostrava, 17. listopadu 2172/15, 70800 Ostrava, Czech Republic; radovan.hajovsky@vsb.cz (R.H.); jan.velicka@vsb.cz (J.V.)

**Keywords:** accelerometer sensor, data processing, IQRF^®^, IQMESH, load anchor cell, monitoring system, protective fence, wireless sensor network

## Abstract

A large number of rock formations in the Czech Republic and abroad directly threaten to damage objects or traffic along the roads located beneath these formations. For this reason, many such rock formations are stabilized using protective fences or dynamic barriers. There are several special sensors available on the market. However, there is no comprehensive monitoring system, including remote threshold settings, data processing, and alarm conditions. This statement is supported by extensive research in this area as well as information from major geotechnical companies that are interested in such a system and want to include it in their portfolio. The aim of the article is to describe the unique wireless monitoring system used to measure the geotechnical quantities we have developed. The design and implementation of systems used to measure protective fence states with accelerometers and slope shift with load anchor cells are presented. Wireless accelerometric sensors and load anchor cell sensors are proposed for both systems. To transfer data from the accelerometer sensor to a superior system, IQRF^®^ technology is applied for the communication between the wireless nodes and the network coordinator under the IQMESH topology. The article includes a detailed description of the development of the accelerometric wireless sensor node and load anchor cell wireless sensor node. Three case studies are also discussed. The first case study focuses on the data implementation and assessment at a testing polygon at the village of Málkov. The second case study describes the data implementation and an assessment of the measuring system under operating conditions in Zbraslav, a municipality near Prague. The third case study describes the implementation and assessment of data from load anchor cell wireless nodes installed in realistic conditions on a supporting gabion wall next to a road. All communication between the sensors and with the IQMESH network coordinator and database was executed wirelessly. The data were archived in a MySQL database and it provides a data source for the assessment and visualizations using the Grafana SW system.

## 1. Introduction

A major problem of installed restraint systems over line structures is the long-term monitoring and reporting of their status. The number of installed systems is growing not only in the Czech Republic but also worldwide. These systems particularly include protective nets [1], protective fences [2], and gabion protective walls [3]. The condition of these restraint systems is usually checked visually, in the presence of the person responsible for the maintenance of the restraint system. However, this method of inspection is very time-consuming and costly. A solution to this problem is to install sensory networks on these restraint systems that will measure the change in their condition and then wirelessly transmit measured data to the dispatching center. This will significantly reduce the cost of visual inspections and will immediately inform the dispatcher of any changes in the status of all the security features [1] where the monitoring systems will be installed [4].

Based on long-term cooperation in the field of R&D with important Czech geotechnical companies and their foreign partners, it was found that there is no commercially available wireless monitoring system enabling the construction of a robust MESH network of sensors, including the possibility of the remote configuration of parameters of individual sensors.

The aim of this paper is to acquaint the reader with the development and implementation of a unique wireless monitoring system designed for the long-term autonomous monitoring of the restraint system over line structures. According to the implementation, the system is designed in two variants. The first is based on the use of accelerometer sensors with wireless data transmission for installation on protective fences. The second is based on the development of a wireless sensor for load anchor cells that measures the movement of protective gabion walls. Both of the systems described are not installed anywhere in the world. This fact is supported by the European leader in the field of installing restraint systems by the company Maccaferri.

The stability of rock formations above line structures and their monitoring is a topical issue that is being addressed worldwide. In particular, twin-screw protective nets [5] in combination with protective fences [2] are used to prevent the stones falling from rock massifs. The paper [5] describes the modeling of a protective net load using LVDT sensors and string potentiometers. The use of these elements for load monitoring has already been considered in [6], but the cost-effectiveness of this solution and the high degree of uncertainty of placing these sensors on these protective networks led us to further develop and implement a system based on accelerometer sensors. The fact that this monitoring system is functional is also demonstrated by the actual application of a similar system for the railways in Scotland, see [7].

One of the possibilities of monitoring the landslide is described by Scaioni in [8]. This review article describes the advances in landslide monitoring with the help of robotic total stations and GNSS (Global Navigation Satellite System) techniques, remote-sensing techniques, such as terrestrial laser scanning (TLS), ground-based SAR (synthetic aperture radar) and satellite differential interferometric SAR. The results of this research are focused on measuring the stability of dams. Scaioni also used TLS methods in [9], where he dealt with graphical methods for processing scanned data to detect rock massif damage. Scaioni described another possibility of rock massif monitoring in [10], where he used a Ground-Based Interferometric Real-Aperture Radar sensor IBIS-S (IDS Company, Pisa, Italy) for the long-term monitoring of quasistatic deformations of these rock massifs. All these methods monitor the rock formations themselves. They make no mention of the monitoring of the actual restraint systems, the condition of which should be monitored on an ongoing basis and at minimum cost. Article [11] describes the application of a combination of Terrestrial Laser Scanning (TLS) and Structure-from-Motion (SfM) photogrammetry to detect rock mass erosion in the Yosemite Valley. Using this method, the authors are able to assess rockfall hazards and provide longer-term rates of cliff erosion.

In article [12], the authors deal with the modeling of shallow landslides caused by collisions using time-lapse electrical resistance tomography (ERT). Time-domain reflectometry (TDR) data were also obtained to obtain volumetric water content. In this study, the authors argue that the application of this technique could be applied to field applications in areas prone to the development of shallow landslides. In our article, we deal with the monitoring of restraint systems that are supposed to retain these erosions and the subsequent rockfall. Nevertheless, it is a suitable gap in the market where further developments in this area could be taken, i.e., linking the prediction of landslides and subsequent monitoring of restraint systems.

The authors of [13] went even further and used a system they called MUNOLD (MUlti-Sensor Network for Landslide Disaster) to monitor landslides. This system is based on the principle of a combination of sensors, including similar sensors and techniques used in [12]. The whole system is the spatial sensor network (SSN), which integrates real-time communication infrastructure and observations from in-situ sensors and remote sensing platforms. This system should be used in places where there is a permanent power supply and the equipment can be protected from the weather and theft. Our designed system does not provide such a comprehensive analysis, but it is characterized by being energy efficient and the size of the sensors and the ease of installation provide a significant advantage. Another parameter is the price of the solution.

As it was previously mentioned, two basic protection systems are used to rehabilitate rock formations. The first type is a high-strength steel network covering the damaged rock mass [1]. The second method is installing protective fences or dynamic barriers [2]. This involves constructing a special fence in which a twin-screw mesh is drawn between the fence rods. This is further reinforced with steel ropes at their edges. Another equally important means to protect roads is the use of gabion walls, which are special iron cages filled with stone [14]. These cages are then bound together. Their primary purpose is to prevent sloped areas from sliding. These slopes require monitoring, and for this purpose, special sensors called load anchor cells may be used.

Different examples of rehabilitation applying barriers and protective networks are described in article [15], which examines the rehabilitation of a rock massif in Germany. Other examples include retaining walls and retaining networks used in Albania [16] or in the USA [3,14]. Reference [2] describes an experiment of monitoring the protective fence using accelerometers. A series of experiments were performed to verify the amplitude of the accelerometer sensors. Accelerometers with 2G resolution were used. Accelerometer data were analyzed using Fast Fourier Transformation (FFT) and Short-Time Fourier Transform (STFT) techniques. However, this article does not describe real installation at specific locations and does not discuss problems that may arise during installation under real conditions, such as power consumption of individual measuring points. There is also no description of the whole monitoring system including the definition of data transfer technology, data archiving, and visualization.

As mentioned here [7] and here [17], a commercial solution based on IQRF^®^ technology works for at least five years in pure battery operation. Another approach to the detection of rock mass destruction and subsequent rockfalls is described in [18]. The detection of rockfalls using infrasound sensors and accelerometers is described here. However, this article is not aimed directly at the use of accelerometer sensors to detect the impact of stone on the restraint system. At present, no comprehensive monitoring system is available to inform network administrators about broken parts of the fences or the amount of fallen rocks captured. Checking these protective fences is conducted only at scheduled times, and the need for the aforementioned type of warnings becomes immediately apparent [4].

This article is an extension of the conference article in [19]. The original article was extended in its assessment of data measured at locations where the applied measuring system is installed. It also incorporates three case studies and includes the new location of Zbraslav. In the introduction, the paper introduces the reader to the solved problems and gives an overview of similarly solved problems in the world. The advantages and disadvantages of existing solutions and innovation of our solution are briefly discussed. In the Materials and Methods section, the authors describe the development of monitoring systems for protective fences and loads on gabion walls. There is a diagram of the complex monitoring system and individual parts of the monitoring system are described in detail. The authors analyze the design of the accelerometer wireless sensor node and load anchor cell wireless node. The use of IQRF^®^ wireless technology for transmitting information from wireless sensor nodes to a network coordinator is also described. There are also three case studies that summarize experiences with the implementation of developed wireless monitoring systems. In the Results section, the authors deal with an analysis and visualization of measured data from individual monitoring systems. The conclusion contains a final summary of the issue and outlines further developments in this area.

## 2. Materials and Methods

This paper describes experimental setups for monitoring the status of protective fences and loads on gabion walls. A method best fitting our requirements for the solution is presented in publication [20], where the researchers describe measuring deflections on a network in a laboratory model. Other publications [21,22] discuss the application of mathematical models of retaining networks and restraining walls on unstable slopes.

**Figure 1:**
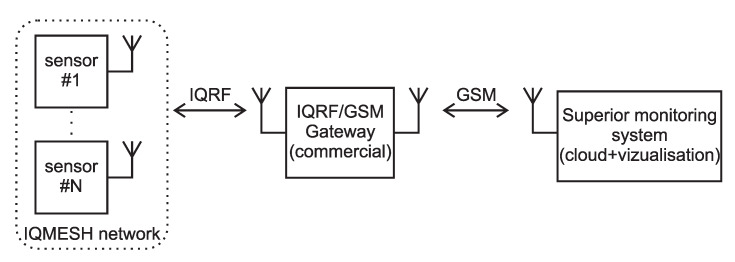
Block scheme of the monitoring system measurement network.

Special measurement networks were proposed for this purpose. The two main components of these measurement networks are the same, consisting of an IQRF^®^/GSM gateway, which collects data from wireless nodes, and a database that permits the visualization of the data. The third part of the measurement network is different according to the type of measurement [23]. A block scheme of the system is shown in Figure 1.

Two nodes with wireless data transfer were developed by the authors for the purpose of monitoring the safety along roads. The first is a simple accelerometer wireless sensor node installed on the protective fence or barrier. The second is a special node that measures electrical resistance at the load anchor cell. This sensor’s electronics are more complex than the accelerometer wireless sensor node. Each component of these wireless nodes is briefly described below.

### 2.1. Accelerometer Wireless Sensor Node

**Figure 2:**
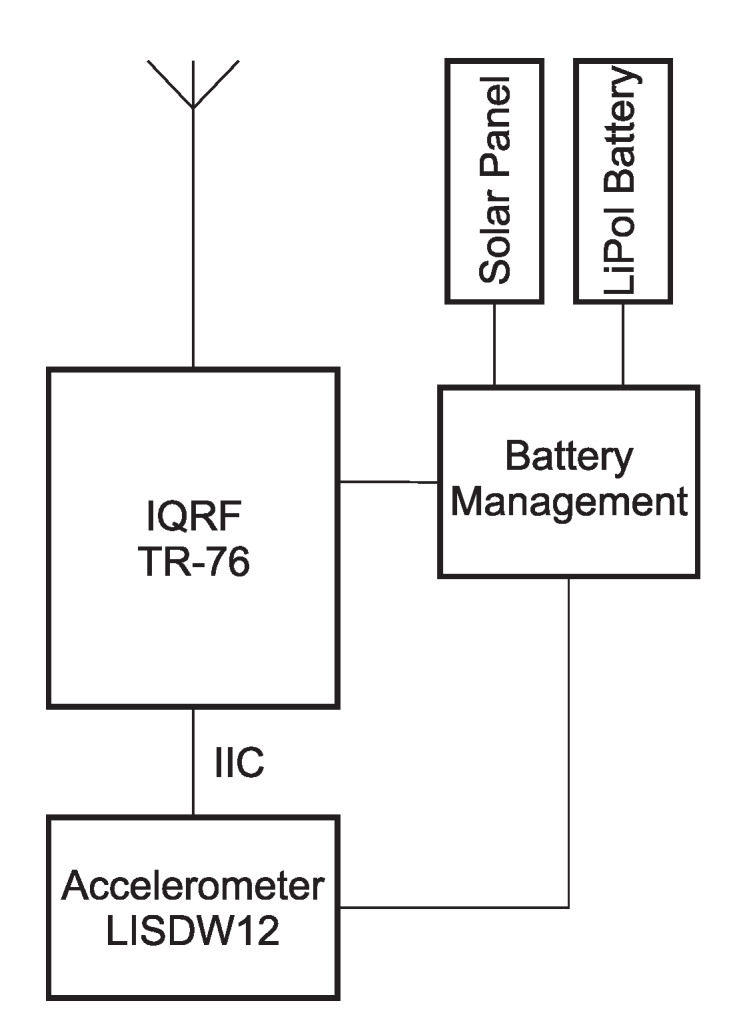
Internal structure of the accelerometer wireless sensor node with the LIS2DW12 sensor.

The internal structure of the accelerometer wireless sensor node is shown in Figure 2. This block diagram of the wireless measurement module shows the links between the blocks.

Three types of accelerometer nodes with wireless data transfer were developed in the course of the last year. The first one (hereinafter referred to as the “first-generation accelerometer node”) used an LIS2DS12 accelerometer sensor. This node did not include circuits for power supply management and was not able to send asynchronous packets. The next two generations of accelerometric wireless sensor nodes used an LIS2DW12 sensor type, which includes circuits for power supply management and can send asynchronous packets. The main difference between the second and the third generations is the size. The third-generation node incorporates a temperature sensor as near to the battery as possible. This accelerometer wireless sensor node can be embedded in a special material to protect the electronics from adverse factors. The third-generation accelerometer node described in the next section of the paper includes wireless data transmission and uses an LIS2DW12 sensor.

The STM LIS2DW12 is an ultra-low-power three-axis MEMS accelerometer. It is very accurate, containing 14-bit analog-to-digital conversion hardware for each channel. It is therefore able to capture the *x*, *y*, and *z* channels simultaneously. This chip also contains an internal temperature sensor, which could be used for temperature compensation in the measured data. The measured data are sent via the IIC bus to the master module, which is the TR-76 IQRF^®^ transceiver. IQRF^®^ technology is described in more detail in Section 2.3.

**Figure 3:**
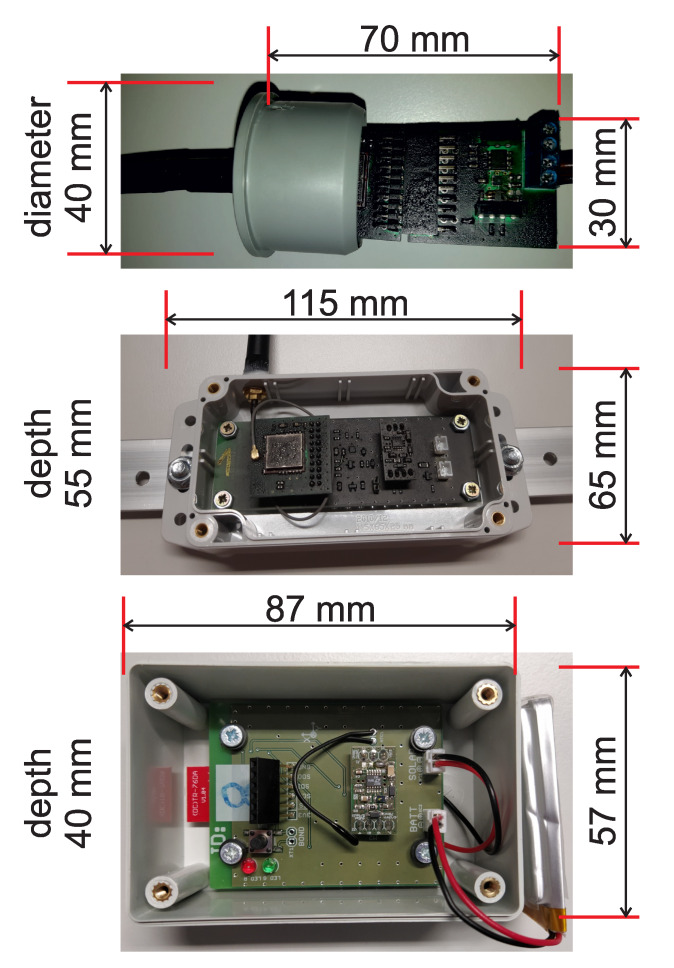
Accelerometer wireless sensor nodes: first generation with LIS2DS12 sensor (**top**), second generation with LIS2DW12 sensor (**center**), third generation with LIS2DW12 sensor (**bottom**).

Figure 3 shows pictures of the three generations of sensors.

The top picture shows the LIS2DS12 accelerometer sensor and its electronics, which were installed into a water-tight plastic tube. The middle and bottom pictures show the LIS2DW12 accelerometer sensor and its electronic components, which were installed into a plastic box. The main difference between these designs is in the use of a solar panel. Older versions (first generation) of the sensor used an external solar panel with a power of 5W and a 3.2Ah LiFePO${}_{4}$ battery. The newer versions use smaller solar panels with 0.5W of power and 2Ah LiPol batteries. The different battery chemistries were considered in the Battery Management block’s design (Figure 2). For this reason, an LT3652 charger with multi-chemistry support was used.

The accelerometer data are evaluated by dividing the data into two components: static and dynamic. The evaluation of steady-state data (static component) is performed so the geometric sum of the components measured by accelerometer sensors must be equal to 1G$\left(9.81\;m/s^{2}\right)$. It is therefore quite easy to separate the measured values into static and dynamic acceleration components. From these measured values it is possible to determine the static change of the position of individual accelerometric sensor nodes and thus to estimate the change of the position of the protective fence. The evaluation of the protective fence dynamic changes is performed so that any change in each component above the set threshold is considered to be an asynchronous event. Accidental incidents, i.e., the impact of a stone on the restraint system, are sent via asynchronous packets, the transmission of which is conditioned by the setting of a threshold that is set by the geological engineer. Multiple sensors are used for this data evaluation. This means that all asynchronous packets are sent to the master system and only here, the evaluation of whether there was a stone impact or a random phenomenon takes place.

### 2.2. Load Anchor Cell Wireless Node

**Figure 4:**
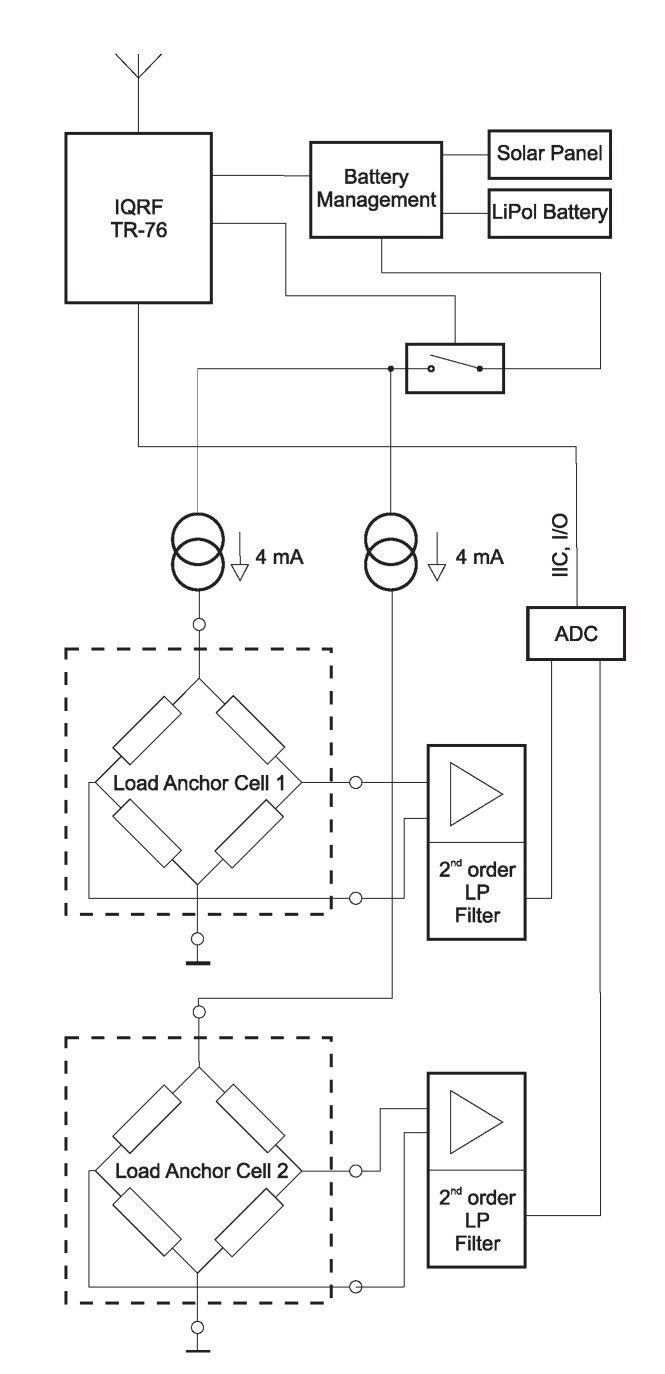
Internal structure of the load anchor cell wireless node developed by the authors.

Each load anchor cell works as a Wheatstone bridge that measures the load at the point where the anchor is installed in the rock mass. The pressure of this massif on this measuring element creates a change in the piezoresistive resistance, resulting in an unbalanced bridge. The internal structure of the load anchor cell wireless node is shown in Figure 4. This block diagram of the wireless measurement module shows the links between the blocks.

To measure the voltage at the load anchor cell, a constant current of 4mA must be provided to the load anchor cell gauge. For this purpose, a precise source of current was used. Each load anchor cell has an input impedance of about 3700Ω on the power terminals; therefore, in order to achieve a constant 4mA current through this bridge, the sensor must be powered with at least 14.8V. Since the entire device is battery-powered, the voltage for these power sources needs to be boosted with a DC/DC inverter. However, this inverter is a source of interference that requires filtering.

**Figure 5:**
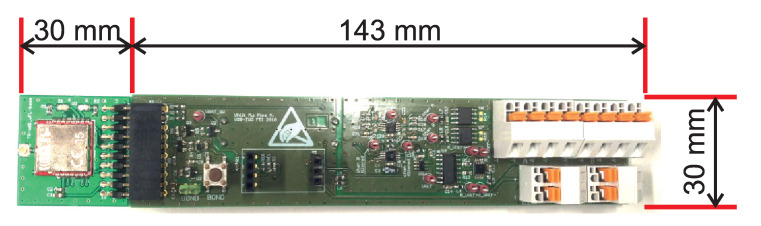
Load anchor cell wireless node developed by the authors, with an IQRF^®^ transceiver.

The measured voltage at the output of the bridge is up to 1000mV. This voltage is produced by an instrumentation operational amplifier and filtered by a second-order low-pass active filter. The instrumentation operational amplifier must be able to accept a higher common voltage on its differential input pins due to high voltage from the current source. The measured voltage is converted with a Σ−Δ 16-bit AD converter. The AD converter communicates with the master module via the IIC bus. The master module is an IQRF^®^ transceiver, which combines an 8-bit microcontroller with an RF chip into a single, small module. Figure 5 shows the IQRF^®^ TR-76 module, on the left side of the image.

The load anchor cell wireless nodes can measure a voltage on two load anchor cells separately. The measured data from the load anchor cell wireless node are transmitted by the IQRF^®^ transceiver with a small mesh topology to the IQRF^®^/GSM gateway.

### 2.3. IQRF^®^ Technology

The IQRF^®^ transceiver module, which is a product from Microrisc, is the main part of both the wireless sensor nodes. In Figure 2 and Figure 4, this module is denoted IQRF^®^ TR-76. IQRF^®^ is a platform for low speed, low power, reliable, and easy-to-use wireless connectivity for telemetry, industrial control, and building automation. IQRF^®^ is a complete ecosystem from one brand and includes hardware, software, development support, and services. IQRF^®^ transceivers can operate in worldwide ISM bands of 433, 868, and 916MHz. The data rate is limited to 19.2kb/s, but for automated data collection from sensors or for controlling actuators in home automation, this is sufficient. The IQRF^®^ consists of an 8-bit microcontroller and RF chip with additional hardware such as an EEPROM memory, LED, and temperature indicator. Several types of IQRF^®^ modules are available. The TR-76 module has a minimum of additional components, which reduces power consumption. Its additional advantage is extra input/output ports. Communication is packet-oriented and allows a maximum payload of 64B per packet. The maximum number of nodes in one IQRF^®^ network is 240 devices, including the coordinator [23,24]. IQMESH is a protocol for wireless IQRF^®^ networks developed by Microrisc. It is based on a mesh topology, which ensures better coverage and a broader range for IQRF^®^ transceivers deployed at a location. Neighbors are transceivers that are mutually within the transmission range.

The article [25] briefly describes the genesis of IQRF^®^ technology and shows a simulation of signal propagation in IQMESH networks in various environments. Finally, it provides a comparison with other wireless communication technologies, such as LoRa WAN, ZigBee, LP BLE, Wi-Fi MESH HaLow, and LoRa WAN. The article [26] gives a comparison of IQRF^®^ technology with LoRa and Sigfox technologies in terms of data throughput and collisions in transmitting messages.

The bandwidth on one IQRF^®^ communication channel is 100 kHz according to [27]. The table in Appendix 2, see [27], describes that the busiest part of the ISM band is at the frequencies of from 869.4 to 869.65 MHz, where the utilization is less than 10% per hour. The default channel No. 52 for communication in the IQMESH network is at a frequency of 868.35 MHz, which shows less than 1% utilization per hour. When installing a new IQMESH network, we use an ISM band scanner to monitor the environment for at least one hour and then select a communication channel with the least data traffic. The table in Appendix 2 shows that at 863.15 to 867.95 MHz, the utilization rate is less than 0.1% per hour, so we prefer to use channels in which the frequencies fall within this area.

Communication in the IQMESH network can naturally result in data loss during communication. In the case of periodic polling, the data loss is treated by a repeated query on the sensory node. In the case of asynchronous packets, there may be crosstalk when multiple sensors are communicating at the same time. However, there is a great advantage of the mesh topology of the sensor communication network we use because a message from the individual accelerometer sensors is sent to the IQMESH network coordinator via [28] “flooding”. At this “flooding”, there is a high probability that the measured data from individual IQRF^®^ nodes will reach the IQMESH network coordinator at least once.

Mesh topology involves connecting each node to all others (full mesh), thereby providing high reliability. If any of the routes become unavailable, an alternative path to deliver messages (usually multiple paths) can therefore be formed. In a wireless IQRF^®^ network using mesh topology, each node has a defined time transmission slot, which ensures transmission does not interfere with other nodes. Nodes continuously receive and forward messages during their transmission slots to all nearby devices. If multiple nodes are somehow damaged, messages have a high probability of arriving at the destination due to the presence of alternative paths. Routing in IQRF^®^ networks is based on directional flooding of the network and TDMA (Time division multiple access) [29]. An example of how the communication among IQRF^®^ nodes works is shown in animation [30]. The main control transceiver the coordinator dispatches data when required. Nearby transceivers receive these data and progressively forward them to the neighborhood during their time intervals. Other transceivers then receive this data and again progressively forward it during their time intervals, spreading the data throughout the network in this manner [24,31].

### 2.4. Energy Requirement Analysis

The energy requirement of the entire measurement network can be divided into three parts.

- The first part is the energy requirement of the sensor module, which waits for the measurement requirement.
- The second part is the energy requirement of the IQRF^®^ communication technology, i.e., the energy requirement of the transmission of information from the sensor module to the IQRF^®^/GSM gateway.
- The third part is the energy requirement of the IQRF^®^/GSM gateway itself.

The energy consumption of the sensor modules is given as the sum of the consumption of the individual components used in each module.

For the accelerometric wireless sensor node, the consumption of key components, see Figure 2, is determined as follows:

- LIS2DW12 accelerometer sensor: 5μA
- Battery Management: 140μA
- IQRF^®^ module TR-76 (operating in low power mode): 250μA

Overall, this is 395μA.

For the load anchor cell sensor node, the consumption of key components, see Figure 4, is determined as follows:

- Current source: (2×4)+0.52=8.52 mA (2 channels of 4 mA + own consumption of integrated circuits making up the current source)
- 2nd order low pass filter: 720μA (both channels)
- AD converter: 150μA
- Battery Management: 140μA
- IQRF^®^ TR-76 module (operating in low power mode): 250μA

Overall, this is 9.78 mA.

It should be noted that the analog part of the load anchor cell sensor node is switchable, i.e., the low pass filters, power sources, and AD converter are active only at the time of measurement where the measurement time lasts two seconds to stabilize transients in the analog part of the electronics. This means that when the load anchor cell sensor node is not measuring, the consumption of the sensor is given by the sum of the TR-76 IQRF^®^ transceiver and battery management, i.e., 390μA.

The energy requirement of the communication technology will be demonstrated as a comparison of the basic transceiver module from Sigfox WISOL SFM10R1 and the TR-76 IQRF^®^ transceiver. More information on comparisons with other technologies can be found in [32].

When communicating with the Sigfox module we consider the following parameters:

- Supply voltage $U_{S}=3.3$ V
- Average consumption $I_{S}=40$ mA
- Payload = 12 B
- Transmission time for one message $T_{S}=2$ s
- Number of repetitions $N_{S}=3$

The resulting energy is then

$$E_{S}=U_{S}\cdot I_{S}\cdot T_{S}\cdot N_{S}/Payload=3.3\times 0.04\times 2\times 3/12=0.066\;\left(\mathrm{Ws}/B\right)$$

When communicating with the TR-76 IQRF^®^ transceiver, the transmission time of a single message varies depending on the payload [33]. With a maximum payload of 64 B, the time to send this message is 100 ms. Summing up this information, we receive the following information:

- Supply voltage $U_{I}=3.3$ V
- Average consumption $I_{I}=25$ mA
- Payload = 64 B
- Transmission time of one message $T_{I}=100$ ms
- Number of repetitions $N_{I}=1$

The resulting energy is then

$$E_{I}=U_{I}\cdot I_{I}\cdot T_{I}\cdot N_{I}/Payload=3.3\times 0.025\times 0.1\times 1/64=0.00012891\;\left(\mathrm{Ws}/B\right)$$

It is clear from these basic calculations that the use of IQRF^®^ technology is significantly better from an energy point of view. Based on this calculation, it can be stated that the TR-76 IQRF^®^ module consumes 500 times less power with five times higher payload. The number of repetitions of messages in the IQMESH network depends on the location of the sensor in the IQMESH network, i.e., the specific network topology [28]. With IQRF^®^ transceivers, the routing algorithm can be turned off and thus made into a terminal device.

The overall energy consumption of the sensor or the entire monitoring system depends on other components and parameters. These include the size of the message being sent in bytes, the period of measurement and messaging, the number of sensors in the IQMESH network, and the number of asynchronous packets sent. The accelerometer wireless sensor node sends 24 B of data and the load anchor sensor node sends 12 B of data. The number of sensors in the IQMESH network and the period of measurement by wireless sensor nodes is determined by the location of the installation based on the geological engineer’s monitoring requirement.

The consumption of the IQRF^®^/GSM gateway is stated by the manufacturer to average 150 mA [34]. This power consumption is reported during a 10-s period of communication with the IQRF^®^ cloud, which allows an almost instantaneous response to occurring alarm events.

Summarizing all the aforementioned facts, it is not possible to precisely compare the energy intensity for the whole monitoring system. Comparing IQRF^®^ technology with Sigfox technology, the overall energy balance of the measurement network is a highly individualized value that differs for each monitoring system implementation. Only the energy balance of the data transmission from the sensor to the gateway can be calculated exactly.

### 2.5. IQRF^®^/GSM Gateway and Cloud

For all case studies, a commercial GW-GSM-02A IQRF^®^/GSM Gateway was used [24]. The gateway sends the measured data to the IQRF^®^ cloud, which offers a communication interface for further data processing. This commercial gateway could be replaced with a Raspberry Pi [35]. To monitor the protective fences and gabion walls, data were collected separately from wireless sensors communicating with each other in a mesh topology.

Data collection was periodic, with a measurement interval of minutes to hours. This measurement time can be set via the IQRF^®^ cloud, whereby setting the appropriate command, a new polling period value can be written into the network coordinator’s EEPROM memory. The network coordinator is a component of the IQRF^®^/GSM gateway. When collecting data, it is necessary to take into account the energy consumption of measurements and data transmission. Although IQRF^®^ technology is characterized by significantly lower power consumption, communication in the GSM network requires much greater transmission power. For this reason, the measuring interval is set by a geotechnical engineer in the range of tens of minutes to units of hours.

**Figure 6:**
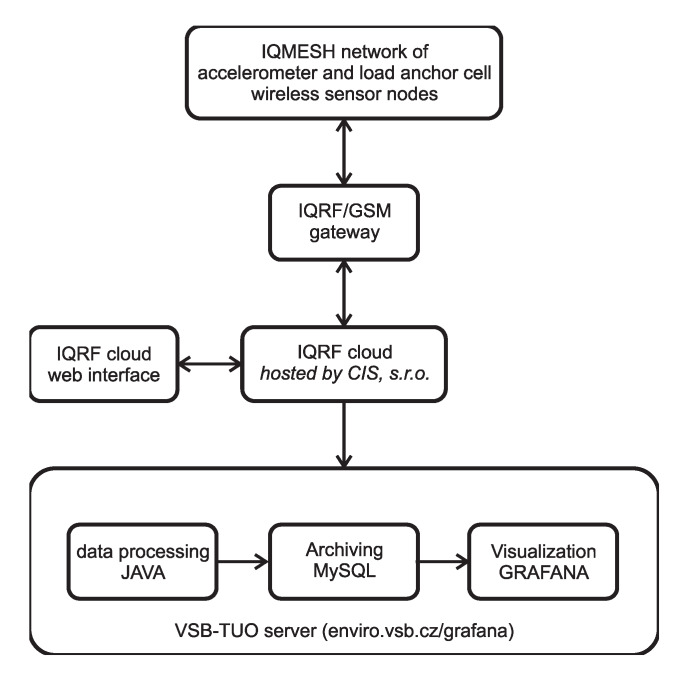
Scheme for processing, archiving, and visualizing measured data for case studies.

The process of data processing and visualization is shown in Figure 6.

**Figure 7:**
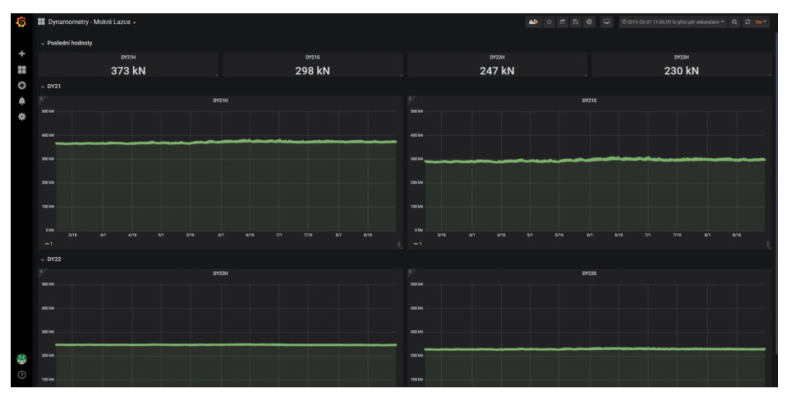
The Grafana system shows the loads on the gabion walls.

As was mentioned, the measured data in raw format are sent to the IQRF^®^ cloud via the IQRF^®^/GSM gateway. The university server enviro.vsb.cz runs a software utility created in the JAVA programming language. This utility queries the IQRF^®^ cloud for measured data cyclically using an HTTP request with a period of 1 min. The raw data are then converted into individual values of measured quantities using this utility and these measured quantities are then stored in the appropriate table in the MySQL database. The database then serves as a data source for visualization using the Grafana SW system. Figure 7 shows an example generated with the Grafana tool. This figure shows the load on the gabion walls between March and mid-August 2019.

### 2.6. Monitoring the Protective Fence

The sensors developed by the authors were installed at three locations. The first location, the village of Málkov, used the first and third generation accelerometer wireless sensor nodes. The first-generation accelerometer wireless sensor nodes continuously measured the static acceleration at equidistant time intervals. The third-generation accelerometer wireless sensor nodes, which were installed on the protective fence as close as possible to the first-generation sensors, measured static acceleration. The sensors were able to send asynchronous packets at the moment the given accelerometer deviated from its balanced state. The second location was Zbraslav, where four second-generation accelerometric wireless sensor nodes were initially installed. They were subsequently replaced by two third-generation accelerometric wireless sensor nodes. This location was used for practical testing of the installation under real conditions. The third location was Mokré Lazce, where load anchor cells were installed on gabion walls.

The paper deals with the complex problem of monitoring the status of protective fences over line structures. In practice, this problem is always resolved by the synergic effect of cooperation between a scientific institution and a commercial geotechnical company. The current installation of sensors depends on the specific location and it is not possible to determine the general instructions on how to place the sensor. The location of the sensor’s installation is always determined by a geotechnical engineer who will assess the most suitable location with regard to possible deformation of the protective fences. The sensor is placed in a plastic box that contains holes for attaching to the protective fence. Usually, the box is fixed by screws and nuts parallel to the protective fence. It can also be attached using special zip ties.

#### 2.6.1. Case Study 1—Installation of the Accelerometer Wireless Sensor Nodes at Málkov

**Figure 8:**
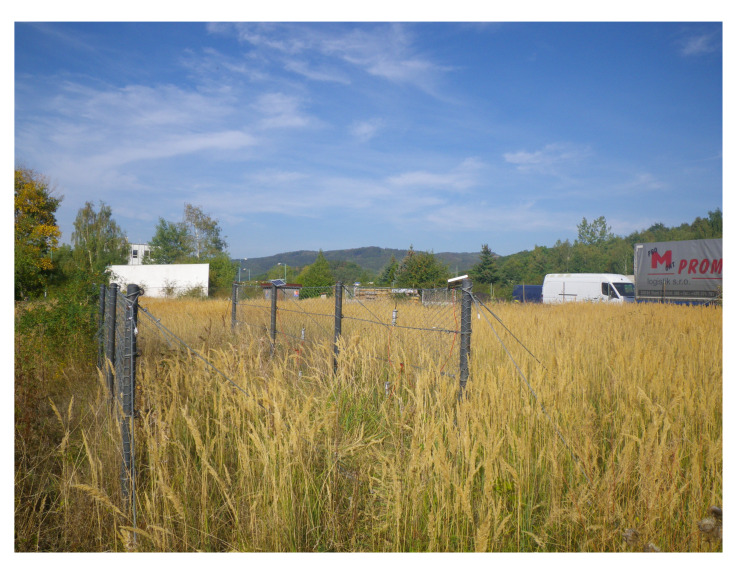
Physical model of the protective fence where the accelerometer wireless sensor nodes were installed.

To evaluate the sensors, a test polygon was created for the village of Málkov. It consists of three fields of protective fences installed onto rock massifs. Figure 8 shows a picture of these fences.

**Figure 9:**
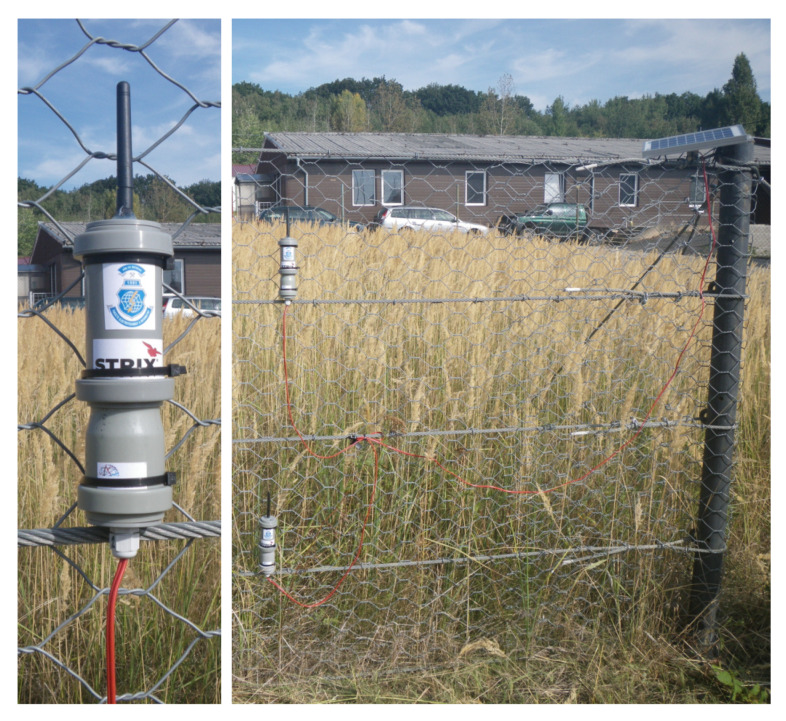
Detailed view of the accelerometer wireless sensor nodes in plastic tubes.

Five first-generation wireless accelerometers were placed in plastic tubes and installed on the test polygon of these protective fences. Four accelerometer wireless sensor nodes were placed on one protective fence, while the fifth was placed on the remaining protective fence. The dimensions of these protective fences are 9 by 1.7 m. These fences are divided into three fields with a length of 3 m. Figure 9 shows the deployment of the two sensors.

A detail of the LIS2DS12 accelerometer sensor, which is in a plastic tube, is at the left of the image. Two of these sensors, which are powered by one 5W solar panel, can be seen on the right.

The initial installation was conducted in order to verify the sensor operation under real and extreme weather conditions, especially in summer and winter. The use of solar panels to charge batteries, their activity throughout the year, and the effect on their efficiency from snow, for example, was also tested. These limit states can be achieved over decades, depending on the type of weathering processes and size of the protective measures. In principle, however, it can be safely stated that achieving such a condition has a high probability [36]. The sensitivity of individual sensors to changes in the protective fence’s status was also tested according to the change’s location. Initial fence stretching tests were described in [37].

**Figure 10:**
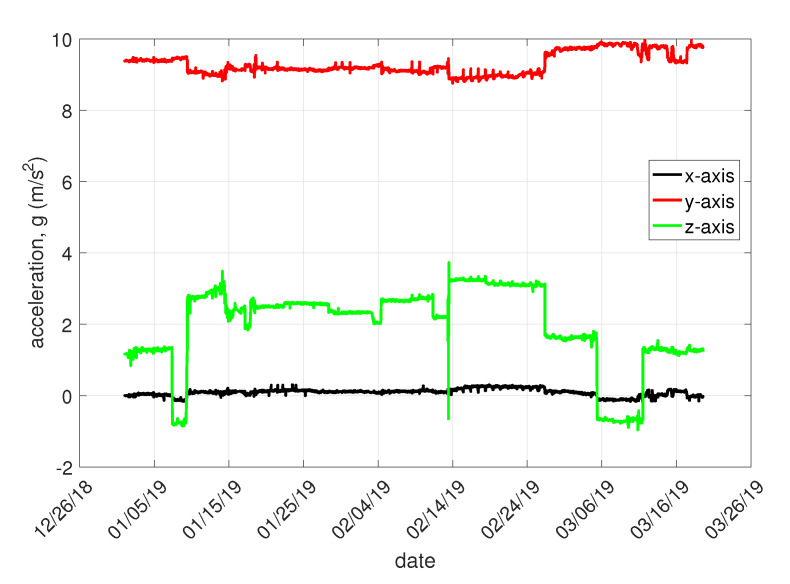
Deflection of the protective fence at the position of accelerometer wireless sensor node No. 1.

The test of the retaining system was conducted from September 2018 continuously at Málkov, a small town in the Czech Republic. Four first-generation accelerometer wireless sensor nodes were tested on the protective fence model. This protective fence was randomly deflected by local workers in order to simulate the possible states of the protective fence. The data in Figure 10 show the gravity acceleration of one sensor from the beginning of 2019 to March in the same year.

The condition of the protective fence was measured at intervals of 30 min. At the time when the load on these fences changed, the measurement interval was set to one minute. As mentioned above, the accelerometer wireless sensor node is based on a three-axis accelerometer. In reality, sensor deflection is measured along the three axes. The vector sum of the individual deflections should be 1G$\left(9.81\;m/s^{2}\right)$. Figure 9 shows that the sensors were placed on the protective fences vertically. Therefore, the displacement at a steady state along the *y*-axis is maximum (approximate. 1). It should therefore be zero along the remaining axes.

Figure 10 shows that this is not the case. Minor inaccuracies from the ideal state were caused by the installation of the sensor, whereas under real conditions it is not possible to guarantee the absolutely accurate mounting of the sensor perpendicularly along the individual planes.

When the status of the protective fence changes, i.e., if it is moved due to, for example, falling stones or rock fragments, the sensor is deflected and the measured value of static acceleration in individual axes therefore changes. Changes are also evident in the visualization graphs: a decrease in values along one axis indicate an increase in values along another axis, maintaining the condition of the sum of individual axes of $9.81\;m/s^{2}$.

Figure 10 shows the progress of the measured data for the accelerometer wireless sensor node No. 1 over the period from the beginning of January 2019, to 20 March 2019. The graph shows the acceleration curves for the *x*, *y*, and *z* axes. During this time period, the monitoring system was tested and the breach of the protective fence was simulated manually. This testing was carried out by pulling the fence in a certain part of the fence. The noticeable deviations from the long-term steady-state acceleration in the individual axes are due to the change in the steady state of the protective fence. Test times were recorded by the person conducting the testing. The dispatching office did not know in advance when the attempt would occur. This was to see if the system was responding correctly to the change of the fence and the data were correctly displayed in the visualization application. After the trial period, the records were then compared and evaluated. An analysis of the obtained data revealed that all simulated failures of the fence were correctly measured and visualized.

#### 2.6.2. Case Study 2—Installation of the Accelerometer Wireless Sensor Nodes at Zbraslav

**Figure 11:**
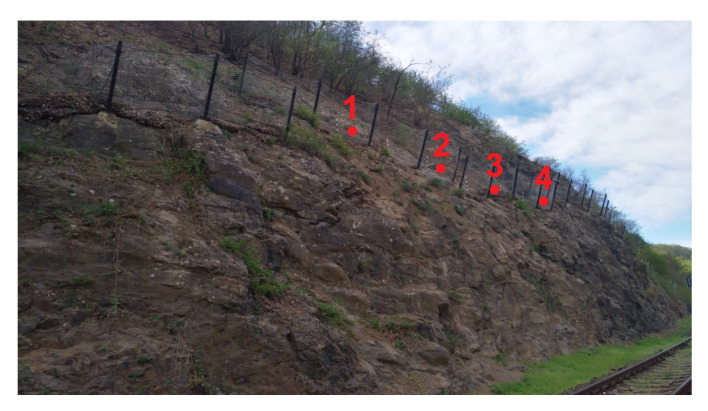
View of the installed protective fences above the railroad.

One of the locations for testing the developed wireless accelerometric sensor nodes was Zbraslav. This area has a section where the installed retaining networks protect a railroad. The area is characterized by a relatively steep rocky slope, which has surface damage and is partially covered with vegetation. This slope immediately adjoins a single-track railroad. Falling rocks and vegetation breaking off and falling is a hazard (Figure 11).

Damaged rock segments of various sizes can be found on the slope, and it is not possible to predict when they will fall. When they do fall, however, they constitute a direct danger to the railroad route below. Vegetation on the slope, particularly trees and their root systems, are unstable due to the damaged bedrock and further contribute to the deterioration of its condition.

**Figure 12:**
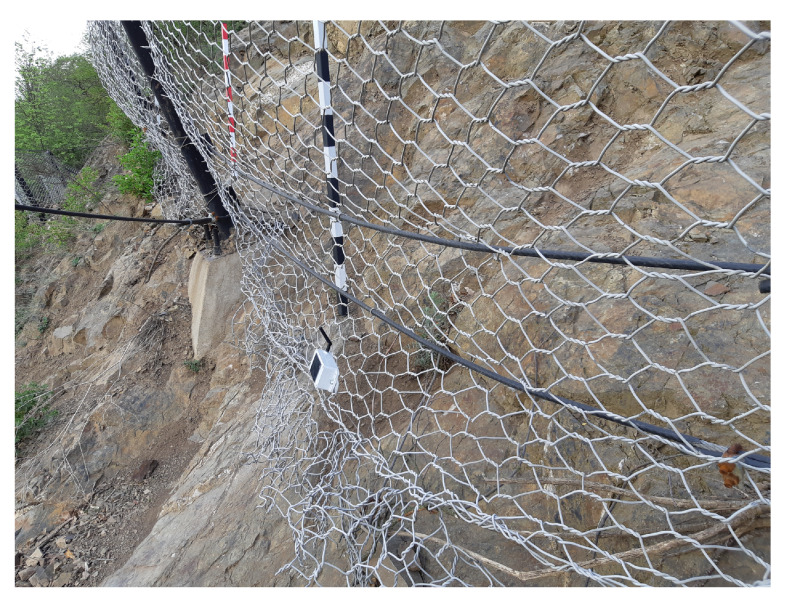
View of the second-generation accelerometer wireless sensor node in the plastic box with solar panel.

Two versions of the accelerometric wireless sensor nodes were tested at this location. In stage one, four wireless second-generation accelerometric wireless sensor nodes, which were bonded (connected) to the IQMESH network coordinator included in the IQRF^®^/GSM gateway, were installed. Figure 11 shows the layout of the sensors. The total length of the monitored section is 20 m; the section is divided into two parts (see Figure 11). The distance between the posts is 3 m. The height of the protective fence is 2 m. The powering accumulators of the wireless sensors were charged using 0.5W solar panels, integrated directly into the sensor installation box. The powering accumulator of the IQRF^®^/GSM gateway was charged using a 40W solar panel. Figure 12 shows the location of this sensor on a real installation.

The IQMESH network coordinator in the IQRF^®^/GSM gateway queried individual network nodes every 12 h. At these moments, data for the measured static acceleration along all three axes were available from all sensors. Between these intervals, asynchronous packets (alarm states) were sent if a specified limit was exceeded. The data were immediately sent to the IQMESH network coordinator if the given static acceleration limit (threshold) was exceeded. This ensured that occurring situations were measured even between the discrete time intervals, in this case every 12 h.

At the end of 2019, the original sensors were replaced with third-generation accelerometric wireless sensor nodes. Two of these sensor types were installed.

### 2.7. Monitoring the Gabion Wall

#### Case Study 3—Installation of the Load anchor Cell Wireless Nodes at Mokré Lazce

The load anchor cells were installed to monitor a slope’s condition. If the slope began to move, it would create a danger to the entire road and passing cars. The measured data from the load anchor cells were therefore monitored remotely, and if a sudden change in the measured values (long-term trend) was observed, the dispatching center was able to provide an immediate warning for the site.

**Figure 13:**
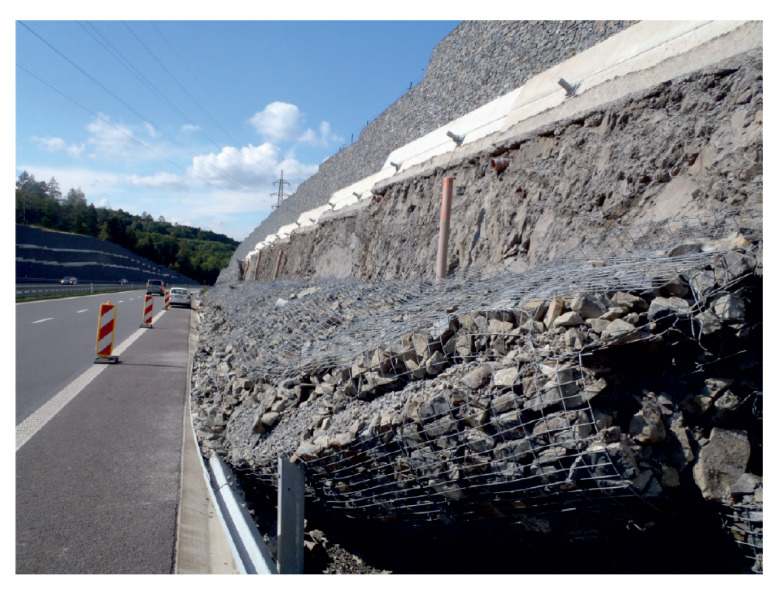
View of the collapsed gabion wall at the given location prior to the installation of the monitoring system.

These slopes are supported by gabion walls. To monitor the condition of these walls, special load anchor cells were installed according to the recommendations of geologists. Figure 13 shows an example of monitoring a hazardous slope condition of a collapsed gabion wall.

**Figure 14:**
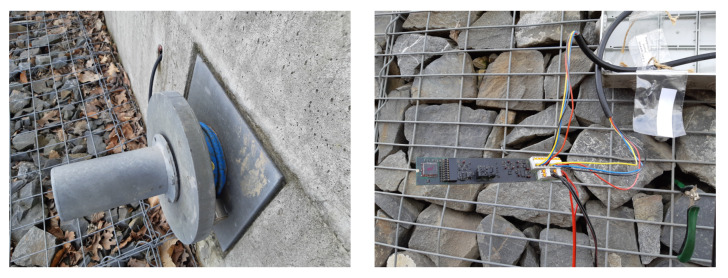
Load anchor cell sensor (**left**), load anchor cell wireless node outside the plastic tube (**right**).

For these reasons, the slope near this hazard was installed with sensors to monitor the condition at the gabion walls. Figure 14 shows the installed load anchor cell sensor (left) and the electronics of the wireless communication load anchor cell wireless node developed by the authors (right).

Four load anchor cell sensors were installed at this location. Since the load anchor cell nodes developed by the authors were 2-channel, it was enough to install two of these nodes to measure the condition of the gabion walls.

The data measured from these sensors were once again sent to the cloud via a commercial IQRF^®^/GSM gateway and then stored in a MySQL database in the same manner described above. Figure 7 shows an example of the load on this wall and changes in the load on the gabion walls between March and mid-August 2019.

**Figure 15:**
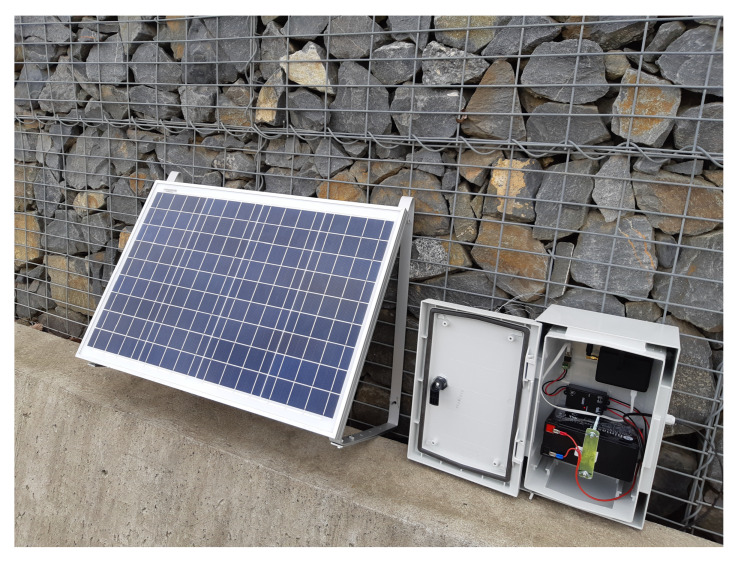
Image of the IQRF^®^/GSM gateway and solar panel installed on the gabion wall.

The system was off grid, i.e., without an external power supply. All components were powered by accumulators charged with solar panels (Figure 15).

Figure 15 shows the installed system for monitoring the gabion walls. The IQRF^®^/GSM, battery and MPPT controller are housed in a robust box (200 mm × 300 mm × 170 mm, IP66). A 40W solar panel with dimensions of 670 mm × 430 mm is located nearby. The whole assembly is located in the middle of the gabion wall, at a height of about 4 m above ground level, making it difficult to steal the entire system. The IQRF^®^/GSM gateway was powered by a 40W solar panel. Each load anchor cell node with wireless data transmission was powered by a 5W solar panel. As mentioned above, each node measured the load of the gabion wall in two positions via two sensors.

## 3. Results

### 3.1. Case Study 1—Málkov

Testing the retaining system was conducted continuously from September 2018 at Málkov, a small town in the Czech Republic. Four first-generation accelerometer wireless sensor nodes were tested on the protective fence model. In 2019, accelerometric third-generation wireless nodes were installed on the testing polygon. They were installed parallel in relation to the previous version of the sensors. The objective of the installation was to determine the behavior of both sensor types from the perspective of long-term off-grid operation and obtain data for their further analysis.

Four accelerometric third-generation wireless nodes were bonded (connected) to the existing IQRF^®^/GSM gateway, thereby changing the existing IQMESH network, which now consisted of nine sensors. The measurements were executed at specified intervals, which could be managed remotely. In this case, queries to the sensors were sent every 30 min. At these moments, data for the measured static acceleration along all three axes from all sensors were sent to the IQRF^®^/GSM gateway. No data for static acceleration were available from the first-generation wireless accelerometer sensor nodes between these times.

This limitation was overcome using the third-generation wireless accelerometer sensor nodes, which are able to send asynchronous packets. The detection technology and the packet sending process are described in article [38]. The data are immediately sent to the IQMESH network coordinator, which is a component of the IQRF^®^/GSM gateway, when the given static acceleration limit (threshold) is exceeded. This ensured that occurring situations were measured even between the discrete time intervals, in this case every 30 min.

**Figure 16:**
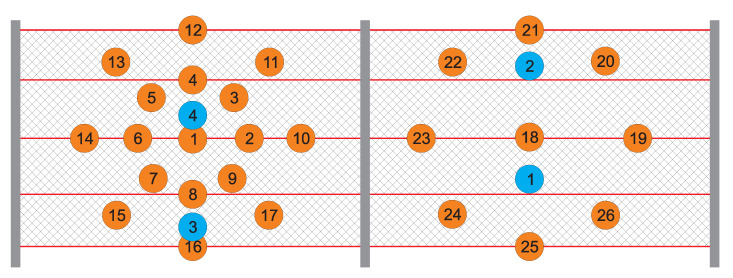
Installation positions on the protective fence of the first-generation accelerometer wireless sensor nodes (blue circles) and network powering locations at the given times (orange circles).

As a part of the experimental testing, measurements on the testing polygon were executed to determine the sensitivity and ability of individual wireless sensors to detect changes in the condition of the protective fence in its surroundings. The condition of the protective fence was manually changed at certain times during these measurements. The acquired data were subsequently analyzed and assessed. The objective of this experiment was to obtain a 3D graph that would demonstrate the relationship between the magnitude of a given protective fence change and the corresponding distance of a given sensor. Figure 16 shows the layout of the sensors and locations where the protective fence was stretched. The orange points, numbered 1 to 26 on the diagram, represent the positions where the protective fence was stretched. The blue points, numbered 1 to 4, represent the positions where the first-generation accelerometer wireless sensor nodes were installed.

Rock impacts were simulated by stretching individual points on the protective fence. Each stretch was done at a certain time. Table 1 gives summarizes the stretch times.

**Figure 17:**
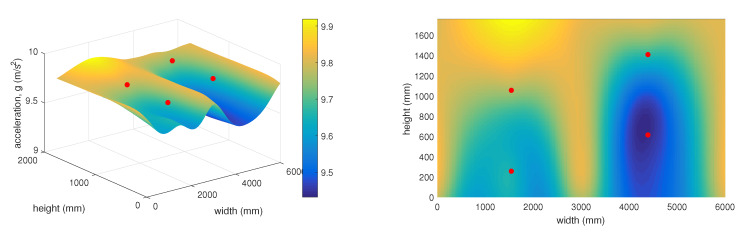
Vector sum of acceleration at 9:48.

The 3D graph in Figure 17 shows the spatial layout of the sensors and changes in the measured static acceleration values along the individual axes in relation to the distances from the simulated rock landing point. This graph represents the time of 9:48, which corresponds to stretch point 6 (Figure 16).

**Figure 18:**
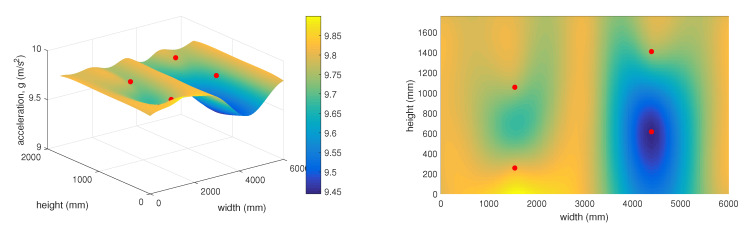
Vector sum of acceleration at 10:48.

The 3D graph in Figure 18 shows the spatial layout of the given sensors and changes in the measured static acceleration values along the individual axes in relation to the distances from the simulated rock landing point. This graph represents the time of 10:48, which corresponds to stretch point 18 (Figure 16).

**Figure 19:**
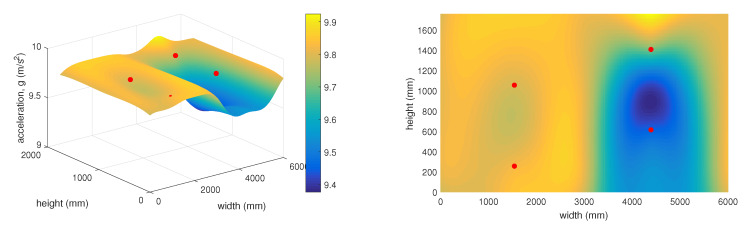
Vector sum of acceleration at 11:23.

The 3D graph in Figure 19 shows the spatial layout of the given sensors and changes in the measured static acceleration values along the individual axes in relation to the distances from the simulated rock landing point. This graph represents the time of 11:23, which corresponds to stretch point 25 (Figure 16).

As mentioned above, the 3D graphs graphically represent the sensitivity of individual surrounding accelerometric wireless sensor nodes in relation to the deflection in the protective fence. This represents falling rocks or other objects into the protective fence under real and practically applied conditions. The objective of the experiment was to determine how stretching the network (a simulated impact of a rock or other object) at a certain point on the protective fence was detected by the surrounding accelerometer wireless sensor nodes. It is clear from the 3D graphs that the accelerometer wireless sensor nodes were highly sensitive and capable of recording changes in the condition of the protective fence even at a significant distance from the point where the loosened material landed. It was also possible to predict the approximate position where the object had landed on the protective fence.

### 3.2. Case Study 2—Zbraslav

The motivation for installing the monitoring system was the requirement for the long-term monitoring of the installed protective fences above the railroad. The protective fences were designed to catch falling rocks and other objects from the slope, which is significantly unstable, and thereby help prevent rocks falling onto the railroad and passing trains. This monitoring system altered the existing system, which operated by obtaining static photographs of the control poles installed at the front of the protective fence. Based on these photographs, the gradually increasing amount of fallen material caught by the fence could be assessed. No information about the rocks that had landed in the protective fence was available prior to the installation of the monitoring system.

**Figure 20:**
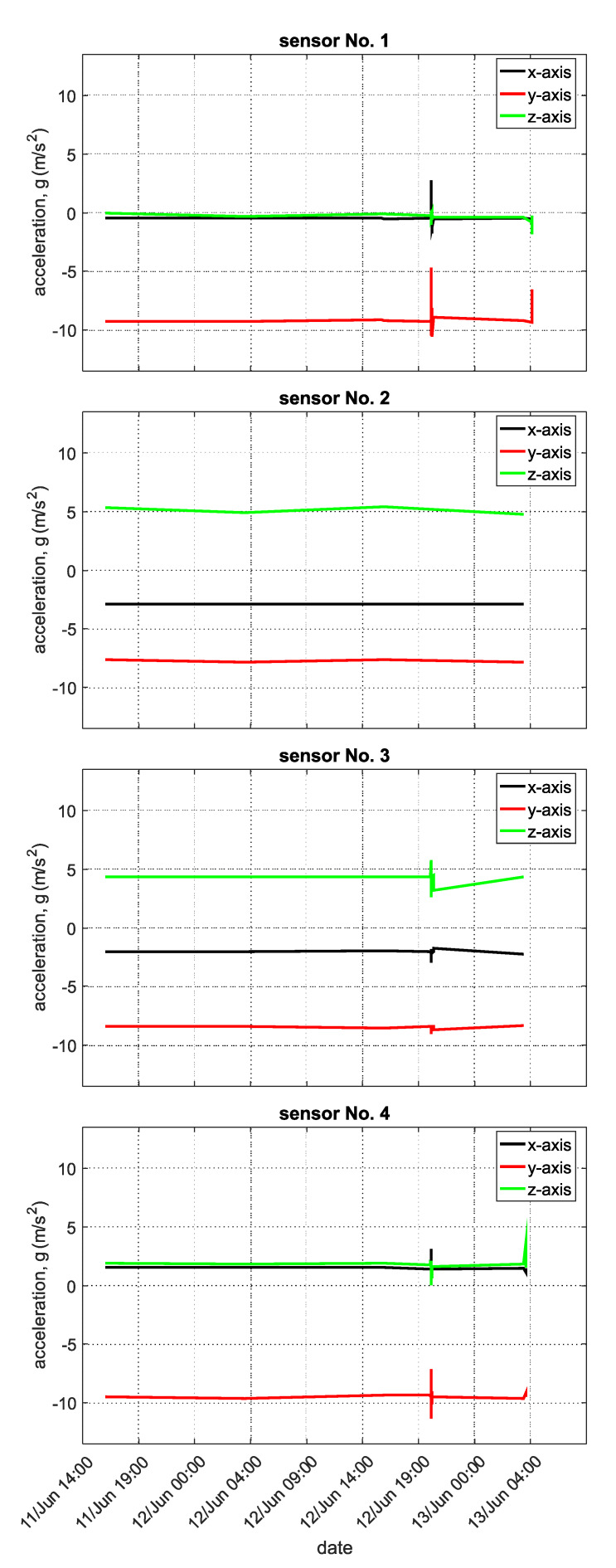
Data from accelerometric wireless sensor nodes installed at Zbraslav.

The measured data show that the asynchronous packets were sent correctly at the given times. The measured data show no changes, and the data time points were recorded every 12 h. By contrast, the time values from the second-generation wireless accelerometer sensor nodes reacted to sudden static acceleration changes (representing changes in the condition of the protective fences), and the data were then apparent even between discrete time intervals. Figure 20 shows the time values from accelerometer wireless sensor nodes installed at Zbraslav.

The graph shows selected time values of the recorded data from a second-generation accelerometer wireless sensor node. The data in Figure 20 were obtained from four accelerometric wireless sensor nodes, of which the installation positions can be seen in Figure 11. It is apparent from the graph that the sensor’s data were obtained at intervals of 12 h. It is also apparent from the graph that two sudden events occurred. These were recorded and sent as asynchronous packets to the IQMESH network coordinator, which is located in the IQRF^®^/GSM gateway. Asynchronous packet generation depends on the size of the acceleration threshold value, which can be set remotely through a cloud service.

The graph in Figure 20 (sensor No. 2) shows a reduced detection sensitivity to the rock impacts, which is why it was not sent to the IQMESH network coordinator.

Figure 20 shows a record of the four accelerometer wireless sensor nodes located on the protective fence (see Figure 11). It can be seen on the record that on 12 June at 20:16 there was an impact on the fence. The impact values were sent as asynchronous packets through the mesh network. It can be estimated that the impact of the stone occurred near wireless accelerometer sensor node No. 1, as shown in Figure 20 above in the *y* axis. Since there were no significant changes in the steady-state values of the individual components of the acceleration, it can be assumed that the protective fence was not destroyed.

The sensitivity of the measurements organized in this manner is convenient, because if a sensor is broken by a falling rock or the asynchronous packet sent to the IQMESH network coordinator is not heard, other second and third-generation sensors remain.

### 3.3. Case study 3—Mokré Lazce

The developed system replaces the original measuring system, which required the encountered forces to be manually measured with load anchor cells. These activities were time consuming and demanding, particularly during winter. The installed wireless sensors allow automated measurements at specified intervals and the transmission of the measured data to a database. The developed visualization application in the Grafana SW system provides appropriately authorized persons with a continuous overview of the currently measured values at the individual load anchor cells. Based on the historical trends and corresponding increases or decreases in stress trends, an authorized person may determine whether the slope is shifting in the monitored area and, if necessary, adopt measures to prevent asset damage and protect humans.

The displayed graphs in Figure 7 show values from four load anchor cells. Taking into account the selected time interval, it is apparent that no changes occurred in the stability parameters of the slope. If a certain part of the slope started collapsing, the load anchor cells would record the corresponding changes in force (and thereby also the stress values). Situations such as this would be immediately evident from the graph.

**Figure 21:**
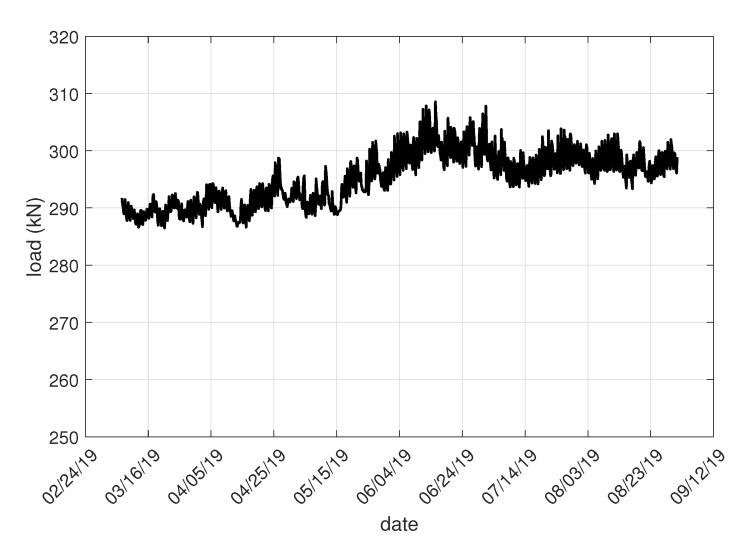
Load at the bottom of the gabion wall.

Figure 21 shows the load at the anchor cell, which is located at the bottom of the gabion wall. The load should be constant. Any deviation would indicate an alarm condition indicating slope movement.

The graph shows that the load on the gabion walls has slightly increased since the beginning of April, but the load trend does not increase or decrease in the following period. This increase in load could be caused by heating of the rock massif and the gabion wall itself. More detailed conclusions can be evaluated after a year of monitoring.

## 4. Conclusions

The paper described the development and implementation of the monitoring system for measuring the state of restraint systems. A system for measuring the condition of protective fences and a measurement system using load anchor cells were proposed and implemented. Both systems were deployed at suitable locations under real operating conditions and described in detail for three case studies, specifically for the Málkov, Zbraslav, and Mokré Lazce locations. The acquired data were archived in a database and visualized using Grafana software.

Based on years of experience in the design and implementation of low-power wireless monitoring systems, the authors have developed three generations of accelerometer sensor nodes with wireless transmission. Some installations already use the newer type (third generation) of the wireless accelerometer sensor node, which uses a much smaller solar panel that can reliably power the sensor. No problems were detected during the operation of the entire system. Charging the powering accumulators using solar panels was tested on both sensor types (accelerometer sensor and load anchor cell sensor). This choice proved to be sufficient, even under adverse climatic conditions. The main advantage of the designed sensors is their capability of sending alarm states using asynchronous packets when specified limit values of measured quantities are exceeded. In the past, measurements involved a person traveling to the sensor and measuring the values at specified times. Transferring data wirelessly will greatly improve the entire measurement network. If an anomaly suddenly occurs, the sensor sends warning information and the dispatching center may respond immediately with an intervention at the relevant location. This can aid in minimizing potential damage to property and hazards to human health. The geotechnical engineer sets the threshold of sending alarm messages from individual sensors (accelerometric wireless sensor nodes and load anchor cell nodes) depending on the location and his experience. A significant advantage is that this threshold can be set remotely based on the verification operation. The successful testing of the sensors in operating conditions confirmed the suitability of the proposed sensors and system for charging the powering accumulators. The massive installation of third-generation accelerometric wireless sensor nodes in cooperation with industrial partners is planned for the future in locations where protective fences have been already installed, especially above line structures. Extending the monitoring system to other locations along roads and utilizing load anchor cell nodes is also planned.

The developed wireless sensors fill the market gap and successfully complement the portfolio of major geotechnical companies with the possibility of implementing wireless monitoring systems based on the use of MESH sensory network topology. The described monitoring system allows the remote monitoring of geotechnical restraint systems. This can prevent significant losses through early warnings of non-standard phenomena—predictive maintenance.

## Figures and Tables

**Table 1:** Stretch times of individual protective fence points.

**Stretching Point**	1	2	3	4	5	6	7	8	9
**Time**	9:23	9:28	9:33	9:38	9:43	9:48	9:53	9:58	10:03
**Stretching Point**	10	11	12	13	14	15	16	17	18
**Time**	10:08	10:13	10:18	10:23	10:28	10:33	10:38	10:43	10:48
**Stretching Point**	19	20	21	22	23	24	25	26	
**Time**	10:53	10:58	11:03	11:08	11:13	11:18	11:23	11:28	

## Abbreviations

The following abbreviations are used in this manuscript:

GW-GSM-02A	Commercial IQRF^®^ GSM Gateway
IQMESH	Type of topology in IQRF^®^ wireless network
IQRF^®^	Trademark name of the wireless communication technology used in the nodes and coordinator in the MESH topology network
LIS2DS12	Name of the accelerometer used in the older type of wireless accelerometer sensor node developed by the authors
LIS2DW12	Name of the accelerometer used in the newer type of wireless accelerometer sensor node developed by the authors
LVDT	Linear variable differential transformer
TR-76	Name of the IQRF^®^ transceiver
VŠB-TUO	Vysoká škola báňská—Tecnická univerzita Ostrava—name of the university
